# A neutralized human LMP1-IgG inhibits ENKTL growth by suppressing the JAK3/STAT3 signaling pathway

**DOI:** 10.18632/oncotarget.14032

**Published:** 2016-12-20

**Authors:** Yuan Mao, Jun Wang, Mingzhi Zhang, Weifei Fan, Qi Tang, Siping Xiong, Xiaojun Tang, Juqing Xu, Lin Wang, Shu Yang, Suyao Liu, Li Xu, Yan Chen, Lin Xu, Rong Yin, Jin Zhu

**Affiliations:** ^1^ Department of Thoracic Surgery, Nanjing Medical University Affiliated Cancer Hospital, Jiangsu Key Laboratory of Molecular and Translational Cancer Research, Cancer Institute of Jiangsu Province, Nanjing, China; ^2^ The Fourth Clinical College of Nanjing Medical University, Nanjing, China; ^3^ Department of Hematology and Oncology, Department of Geriatric Lung Cancer Laboratory, Jiangsu Province Geriatric Hospital, Nanjing, China; ^4^ Department of Oncology, The First Affiliated Hospital of Zhengzhou University, Zhengzhou, China; ^5^ Department of Pathology and The Key Laboratory of Antibody Technique of Ministry of Health, Nanjing Medical University, Nanjing, China; ^6^ Department of Pathology, Jiangsu Cancer Hospital, Nanjing, China; ^7^ Huadong Medical Institute of Biotechniques, Nanjing, China

**Keywords:** LMP1, IgG, ENKTL, JAK/STAT

## Abstract

Latent membrane protein 1 (LMP1), which is associated with the development of different types of Epstein-Barr virus (EBV) related lymphoma, has been suggested to be an important oncoprotein. In this study, a human anti-LMP1 IgG antibody (LMP1-IgG) was constructed and characterized by ELISA, western blotting (WB), affinity and immunohistochemistry (IHC) analyses. CCK-8, MTT, apoptosis assays, antibody-dependent cell-mediated cytotoxicity (ADCC) and CDC (complement-dependent cytotoxicity) assays were performed to evaluate the inhibitory effects of LMP1-IgG on extranodal nasal-type natural killer (NK)/T-cell lymphoma (ENKTL). Then, the influence of LMP1-IgG on the JAK/STAT signaling pathway was investigated. The results showed that the successfully constructed LMP1-IgG inhibited proliferation, induced apoptosis, and activated ADCC and CDC of ENKTL in a concentration- and time- dependent manner. Moreover, phosphorylation of JAK3 and STAT3 was inhibited by LMP1-IgG. Our data indicate that LMP1-IgG may provide a novel and promising therapeutic strategy for the treatment of LMP1-positive ENKTL.

## INTRODUCTION

Extranodal nasal-type natural killer (NK)/T-cell lymphoma (ENKTL) is a subgroup of non-Hodgkin lymphomas (NHLs) characterized by progressive necrotic lesions in the nasal cavity and/or extranasal sites [1–3]. Epidemiologically, ENKTL accounts for 3–8% of malignant lymphomas in China and is more prevalent in Asian than in Western countries [4]. Clinically, ENKTL is highly aggressive and critically difficult to heal, with a median overall survival of less than 8 months [5]. In ENKTL, recurrent drug resistance and immune suppression are common, and the prognosis of ENKTL patients for whom initial therapy fails is tremendously poor [6]. Currently, the lack of any established therapy protocols for ENKTL patients presents a major obstacle for ENKTL treatment [7]. There continues to be an urgent demand for innovative and effective therapeutic strategies to treat ENKTL.

The tumorigenesis of ENKTL is highly associated with Epstein-Barr virus (EBV) infection [8]. Latent membrane protein 1 (LMP1), a substantial oncoprotein encoded by EBV, has been suggested to have multiple malignant functions in the development and progression of EBV-related ENKTL [9, 10]. Identification of LMP1 expression is drawing attention as a favorable target for ENKTL treatment [11, 12]. In our previous researches, we have reported the prognostic characteristics of LMP1 in lymphoma and have generated an anti-LMP1 Fab antibody (LMP1-Fab), exerting potential anti-tumor activity in nasopharyngeal carcinoma (NPC) [13–18]. Because of the remarkable relationships between LMP1 expression and ENKTL properties, an anti-LMP1 antibody should bring positive consequences in ENKTL treatment.

In this present study, we developed a human anti-LMP1 IgG antibody (LMP1-IgG) based on the earlier LMP1-Fab antibody. Then, we tested the characteristics and the anti-cancer efficiency of LMP1-IgG in ENKTL. Moreover, we explored the potential mechanism by which LMP1-IgG inhibits ENKTL development.

## RESULTS

### Construction, expression and purification of LMP1-IgG

**Figure 1:**
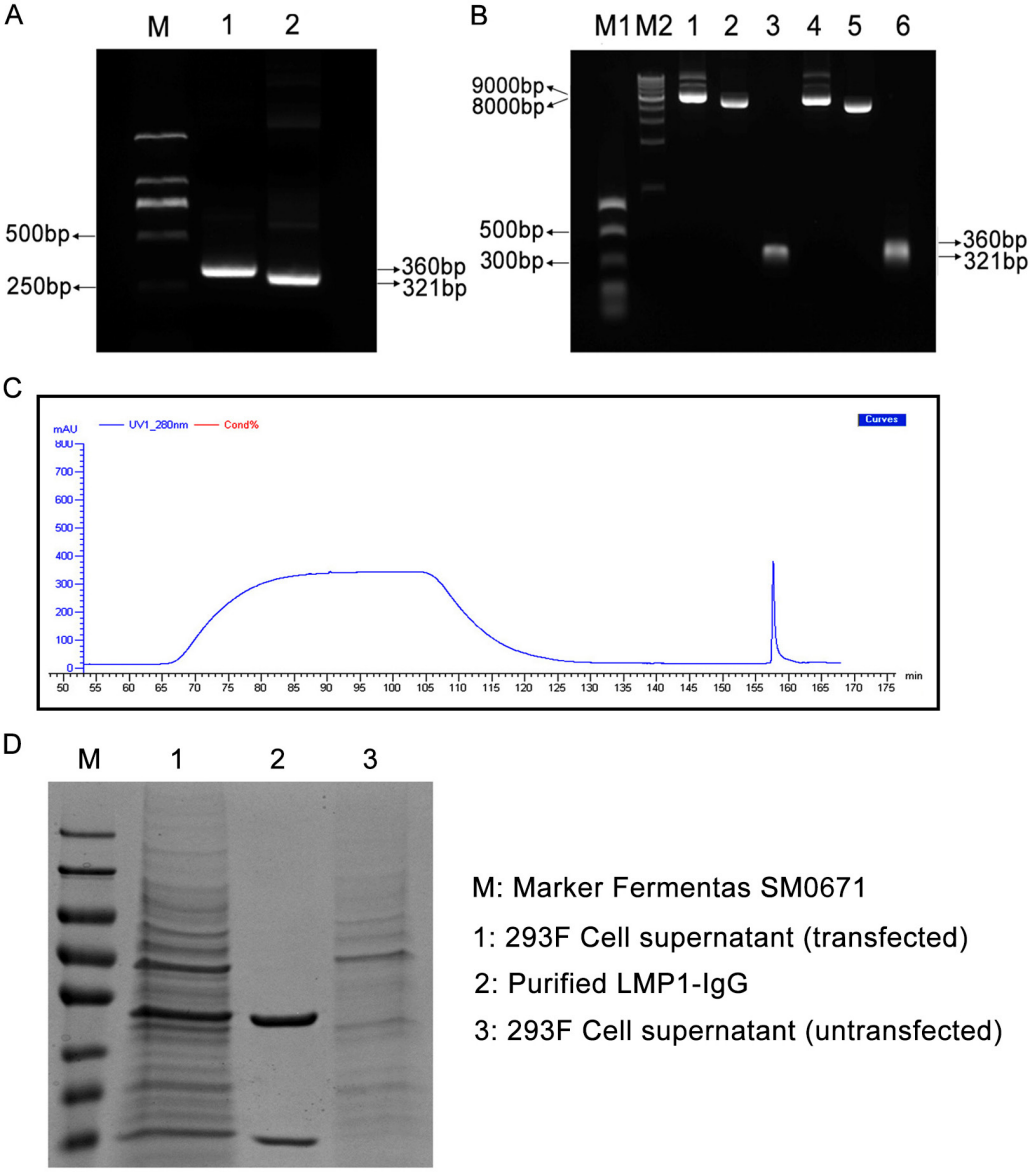
(**A**) LMP1-VH and LMP1-VK variable regions were gathered from a previous LMP1-Fab clone. M: Marker DL2000; Lane 1: LMP1-VH variable region (360 bp); Lane 2: LMP1-VK variable region (321 bp). (**B**) Two recombinant eukaryotic expression vectors (pTH-VH and pTH-VK) were double digested and joined with LMP1-VH and LMP1-VK by Infusion-PCR (IF-PCR). M1: NEB PCR Marker; M2: NEB 1 Kb DNA ladder; Lane 1: pTH-LMP1-VK; Lane 2: Linearized pTH-VK; Lane 3: LMP1-VK; Lane 4: pTH-LMP1-VH; Lane 5: Linearized pTH-VH; Lane 6: LMP1-VH. (**C**) UV curve of LMP1-IgG purification. (**D**) SDS-PAGE confirmed the purification of LMP1-IgG. M: Marker Fermentas SM0671; Lane 1: 293F Cell supernatant (transfected); Lane 2: Purified LMP1 IgG; Lane 3: 293F Cell supernatant (untransfected).

The LMP1-VH (360 bp) and LMP1-VK (321 bp) variable regions were successfully obtained from a previous LMP1-Fab clone (Figure 1A). Two eukaryotic expression vectors (pTH-VH and pTH-VK) were double digested and joined with LMP1-VH and LMP1-VK by IF-PCR separately (Figure 1B). Then, the two recombinant vectors (pTH-LMP1-VH and pTH-LMP1-VK) were transfected with a FreeStyle™ 293 Expression System, and the cell supernatant was harvested. Finally, LMP1-IgG was purified and confirmed with SDS-PAGE (Figure 1C and 1D).

### Characterization of LMP1-IgG

**Figure 2:**
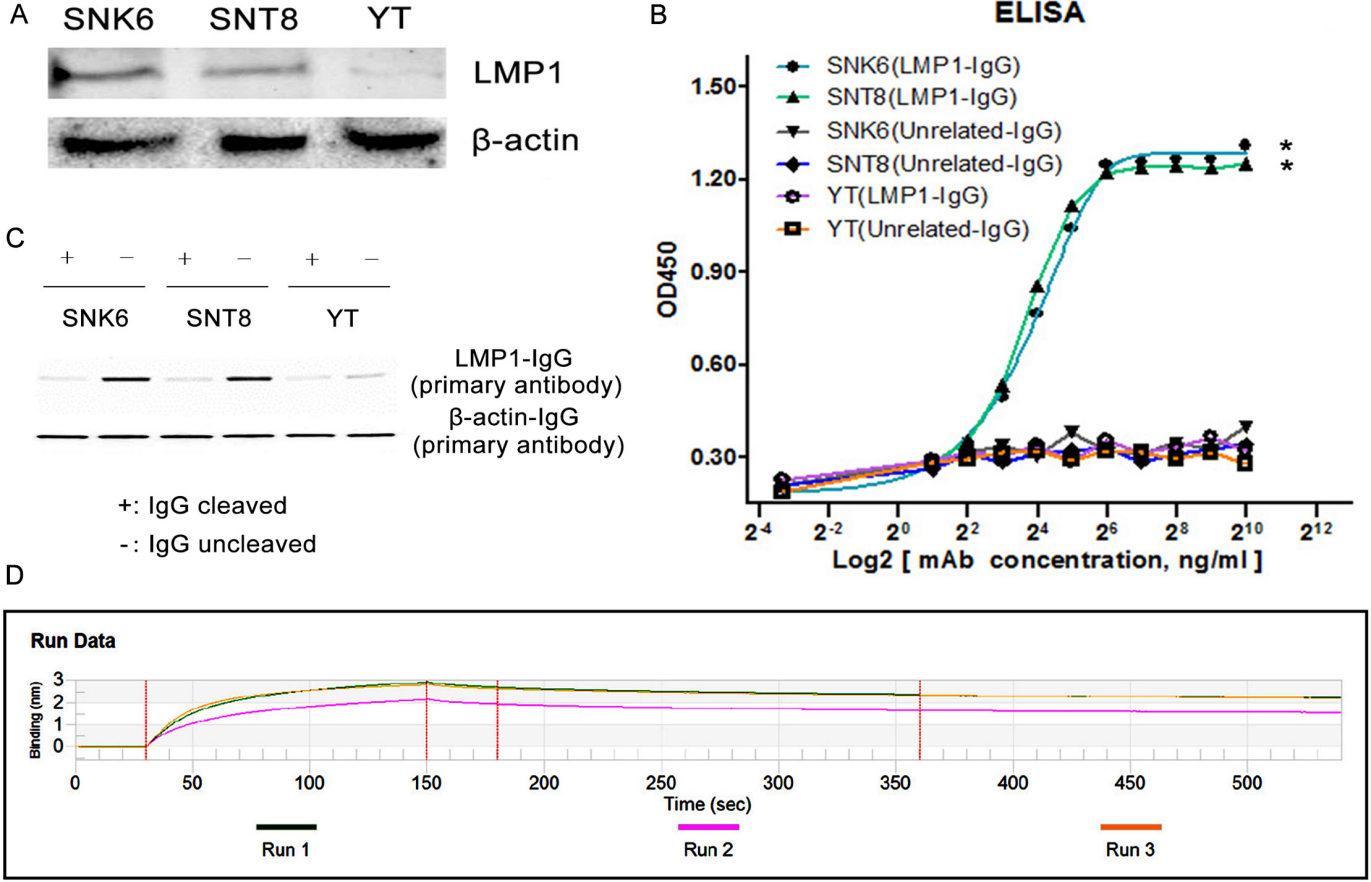
(**A**) WB test confirmed positive LMP1 expression (SNK6 and SNT8 cells) and negative LMP1 expression (YT cells) in three ENKTL cell lines. (**B**) SNK6, SNT8 and YT cells were incubated with LMP1-IgG. SNK6 and SNT8 cells were LMP1-positive; YT cells were LMP1-negative. Unrelated-IgG was used as a negative control. LMP1-IgG specifically reacts with SNK6 and SNT8 cells in a concentration-dependent manner, but not with YT cells. *Indicates significant difference. *p* < 0.05. (**C**) WB test showed LMP1-IgG (uncleaved) recognized LMP1 expressed in SNK6 and SNT8 cells. In comparison, LMP1-IgG was cleaved by papain enzyme and did not recognize LMP1. For LMP1 detection, the primary antibody was LMP1-IgG, and the secondary antibody was anti-human Fc-HRP IgG; for β-actin detection as a control group, the primary antibody was mouse anti-human β-actin IgG and the secondary antibody was HRP-conjugated anti-mouse Fab (Figure 2B). (**D**) An affinity assay demonstrated that LMP1-IgG possessed high affinity for LMP1.

LMP1 expression in ENKTL cells (SNK6, SNT8 and YT) was firstly detected. The information of Figure 2A confirmed positive LMP1 expression in SNK6 and SNT8 cells. In comparison, negative LMP1 expression was observed in YT cells. ELISA was further performed to test the binding sensitivity of LMP1-IgG to LMP1. As shown in Figure 2B, LMP1-IgG recognized LMP1, which was expressed in SNK6 and SNT8 cells in -dependent manner, and the absorbance values of LMP1-IgG in LMP1-positive and -negative cells differed significantly. WB testing showed that LMP1-IgG (uncleaved) could recognize LMP1 which expressed in SNK6 and SNT8 cells. In comparison, LMP1-IgG was cleaved by the papain enzyme and failed to recognize LMP1 (Figure 2C). An affinity assay suggested that the LMP1-IgG possessed a high affinity for LMP1. The equilibrium dissociation constant (Kd) for LMP1-IgG was 3.175 × 10^–9^M (Figure 2D).

### IHC analysis

**Figure 3:**
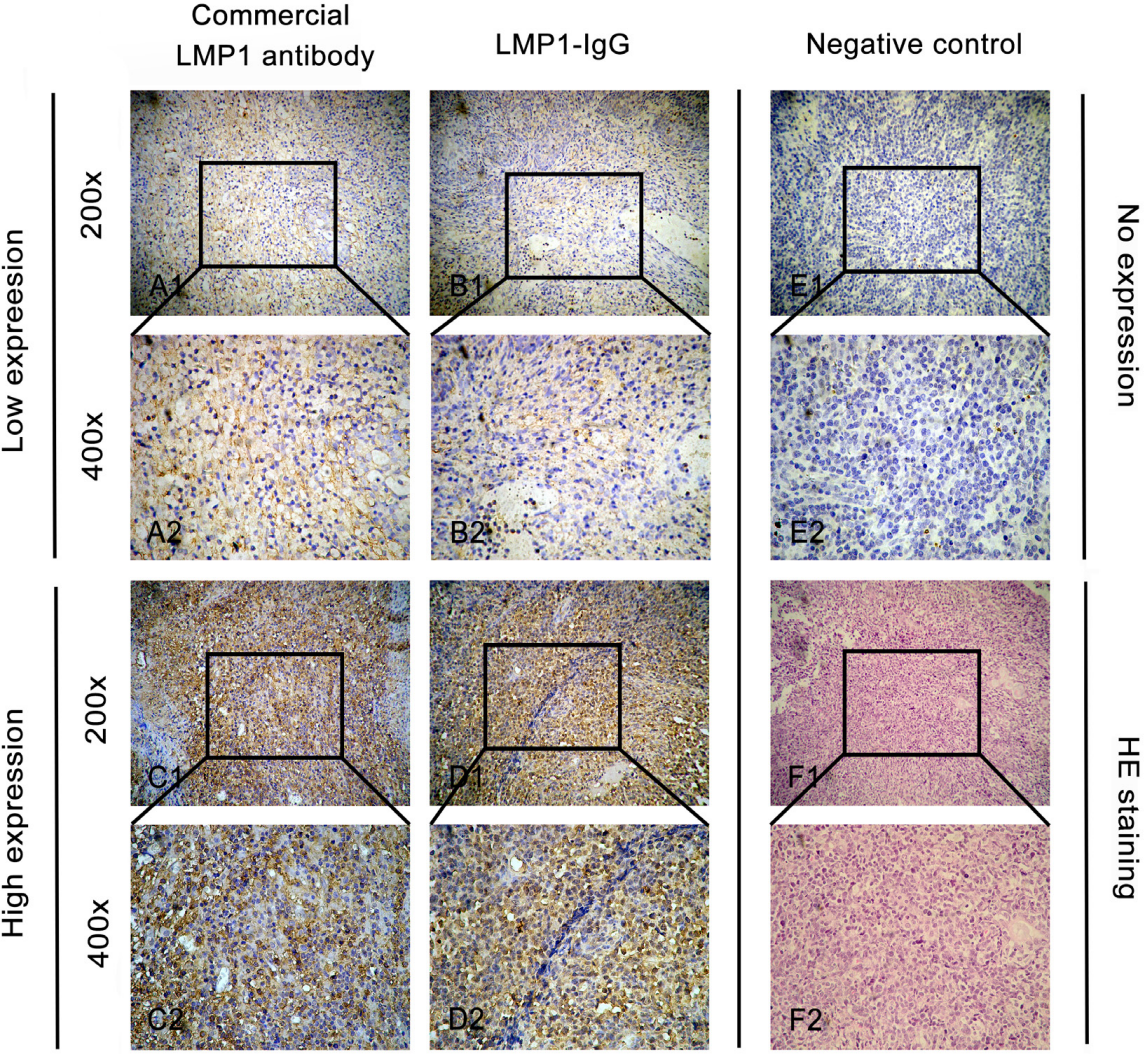
Immunohistochemistry (IHC) analysis in clinical ENKTL samples A1 and A2. Low expression of LMP1 when using a commercial LMP1-antibody as the primary antibody in IHC analysis. B1 and B2. Low expression of LMP1 when using LMP1-IgG as the primary antibody in IHC analysis. C1 and C2. High expression of LMP1 when using a commercial LMP1-antibody as the primary antibody in IHC analysis. D1 and D2. High expression of LMP1 when using LMP1-IgG as the primary antibody in IHC analysis. E1 and E2. Negative expression of LMP1 when using phosphate-buffered saline (PBS) in IHC analysis as a negative control. F1 and F2. Hematoxylin-eosin (HE) staining of ENKTL samples. Original magnification: × 200 in A1, B1, C1, D1, E1 and F1; ×400 in A2, B2, C2, D2, E2 and F2.

IHC was performed in ENKTL tissue samples to further confirm the ability of LMP1-IgG to detect LMP1 expression in clinical samples. A commercial LMP1 antibody (C-LMP1) was used as a positive control. As shown in Figure 3, LMP1 expression was observed in 16/26 (61.5%) cases in the LMP1-IgG group and 18/26 (69.2%) cases in the C-LMP1 group. The LMP1 expression was barely different between the LMP1-IgG and C-LMP1 groups, thus indicating the comparable ability of LMP1 to be recognized by LMP1-IgG and C-LMP1.

### LMP1-IgG decreases ENKTL cell viability and induces apoptosis

**Figure 4:**
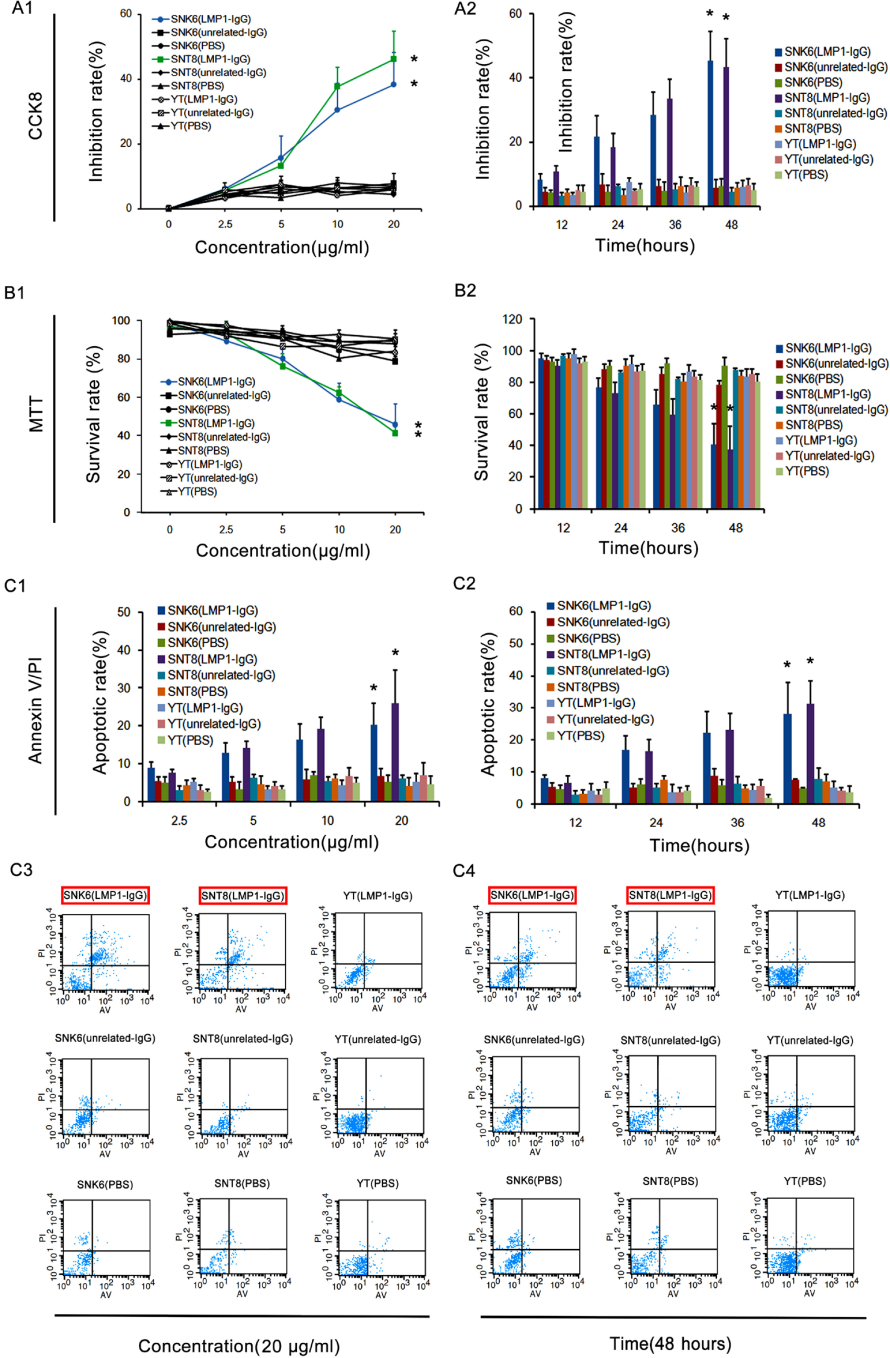
LMP1-IgG inhibits proliferation and induces apoptosis of ENKTL cells (**A1, A2, B1** and **B2**) CCK8 and MTT assays exhibited the concentration- and time-dependent inhibitory effects of LMP1-IgG (2.5–20 μg/ml or 12–48 h treatment) on the proliferation of SNK6 and SNT8 cells, whereas the inhibitory effect on YT cells was low and insignificant. * Significant difference in SNK6 and SNT8 cells with LMP1-IgG (20 μg/ml or 48 h treatment) compared with PBS treatment. *p* < 0.05. (**C1** and **C2**) Apoptotic rates in ENKTL cells treated with LMP1-IgG (2.5–20 μg/ml or 12–48 h treatment). *Significant differences in apoptotic rate in SNK6 and SNT8 cells with LMP1-IgG (20 μg/ml or 48 h treatment) compared with PBS treatment. *p* < 0.05. (**C3**) Representative images of cell apoptosis, detected with flow cytometry by Annexin V/PI double staining after treatment with LMP1-IgG (20 μg/ml). (**C4**) Representative images of cell apoptosis, detected with flow cytometry by Annexin V/PI double staining after treatment with LMP1-IgG (48 h treatment). The red frame illustrates the significantly increased apoptotic rate of SNK6 and SNT8 cells treated with LMP1-IgG.

To determine the tumor inhibitory effect of LMP1-IgG, we determined SNK6 and SNT8 cell proliferation by using CCK-8 and MTT assays, respectively. Cells were cultured in medium with 2.5, 5, 10 or 20 μg/ml of LMP1-IgG for 12, 24, 36, or 48 h. In both CCK-8 and MTT assays, at 20 μg/ml LMP1-IgG after 48 h of incubation, the growth of SNK6 and SNT8 cells was significantly decreased compared with that of YT cells (Figure 4A1, A42, 4B1, 4B2), a result suggesting that LMP1-IgG suppresses ENKTL growth. The IC50 of LMP1-IgG in SNK6 and SNT8 cells was 7.421 μg/ml and 17.68 μg/ml, respectively. To determine whether LMP1-IgG could induce cell apoptosis in ENKTL cells, we performed an Annexin V/PI assay. The results showed significant increases in the apoptotic rates of SNK6 and SNT8 cells in a concentration- and time- dependent manner after treatment with LMP1-IgG. By contrast, the apoptotic rate in YT cells was low and scarcely changed after LMP1-IgG treatment (Figure 4C1–4C4).

### LMP1-IgG activates ADCC and CDC

**Figure 5:**
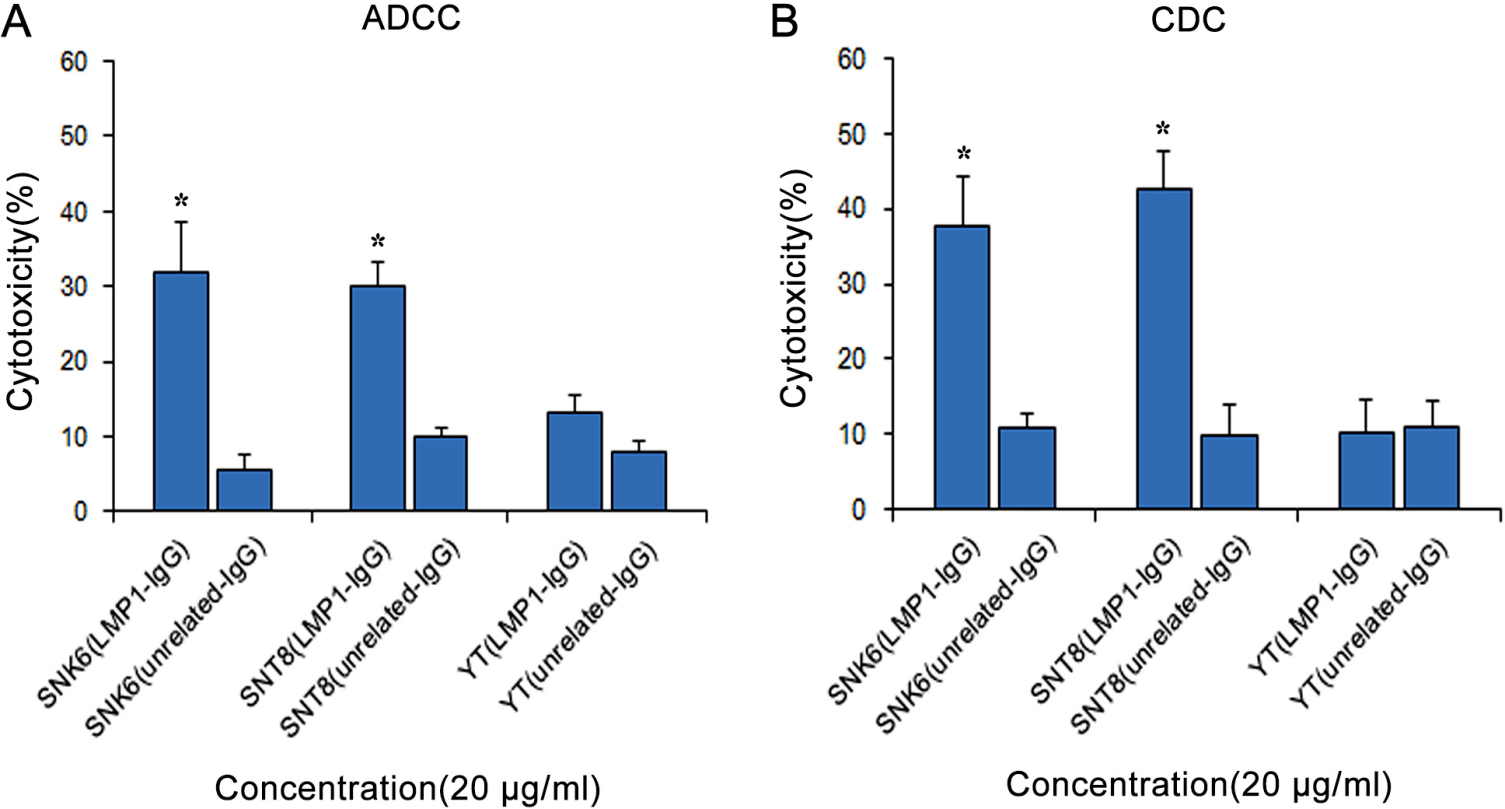
LMP1-IgG activates ADCC and CDC LMP1-IgG (20 μg/ml) induced cell death via ADCC (**A**) and CDC (**B**) in SNK6 and SNT8 cells, but not in YT cells. In comparison, unrelated-IgG barely initiates ADCC and CDC effects. *Significant differences in ADCC and CDC in SNK6 and SNT8 cells with LMP1-IgG (20 μg/ml) compared with unrelated-IgG treatment. *p* < 0.05.

Compared with Fab antibodies, IgG antibodies are theoretically able to induce cell death via both ADCC and CDC mechanisms; hence, we investigated the ADCC and CDC effects of LMP1-IgG. As shown in Figure 5A and 5B, as the concentration increased, LMP1-IgG triggered cell death via ADCC and CDC in SNK6 and SNT8 cells, but not in YT cells. In comparison, an unrelated IgG did not produce ADCC and CDC effects.

### LMP1-IgG inhibits JAK3/STAT3 signaling in ENKL cells

**Figure 6:**
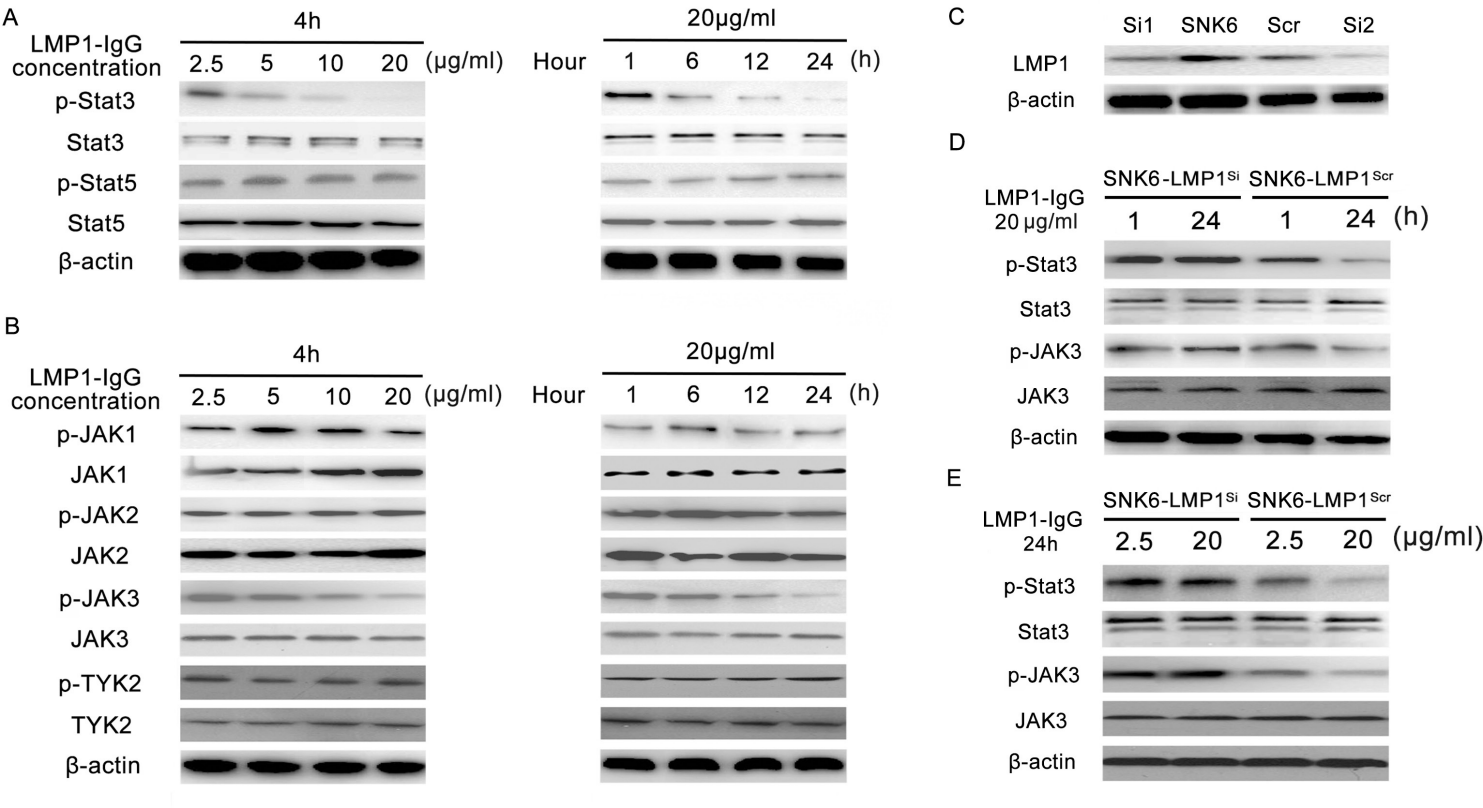
The influence of LMP1-IgG on the JAK/STAT pathway in ENKTL (**A**) Phosphorylation of STAT3 was substantially inhibited after LMP1-IgG treatment in SNK6 cells in a concentration- and time-dependent manner. In contrast, phosphorylation of STAT5 was rarely changed. (**B**) Detection of activation (phosphorylation) levels of the relevant tyrosine kinases in the Janus family (JAKs). LMP1-IgG inhibited the phosphorylation of JAK3 in a dose- and time-dependent manner, but rarely influenced that of JAK1, JAK2 and TYK2. (**C**) Two LMP1-siRNAs and a control-scrambled siRNA were constructed, and LMP1 expression in SNK6 cells was successfully inhibited. LMP1-siRNA2 showed better inhibitory effectiveness for LMP1 expression and was chosen for subsequent experiments. (**D**) and (**E**) LMP1 expression knockdown attenuated the inhibition of phosphorylation of JAK3 and STAT3 (SNK6-LMP1^Si^). In comparison, significant inhibition of phosphorylation of JAK3 and STAT3 was apparent in the control siRNA group (SNK6-LMP1^Scr^).

Since the JAK/STAT signaling pathway is a key molecular factor of ENKTL, we further analyzed the effects of LMP1-IgG on the proliferative and survival signals of JAK/STAT in ENKTL. SNK6 cells were treated with LMP1-IgG at different concentrations and incubation times. As shown in Figure 6A, phosphorylation of STAT3 was significantly inhibited after LMP1-IgG treatment in SNK6 cells in a concentration- and time-dependent manner. In comparison, phosphorylation of STAT5 was rarely changed.

Phosphorylation of STAT3 can be induced by tyrosine kinases of the Janus family (JAKs); we subsequently treated SNK6 cells with LMP1-IgG to examine the activation (phosphorylation) levels of the relevant tyrosine kinases. As shown in Figure 6B, LMP1-IgG inhibited the phosphorylation of JAK3 in a dose- and time-dependent manner, but it rarely influenced that of JAK1, JAK2 and TYK2.

Moreover, we tried to knockdown the LMP1 expression in SNK6 cells and the phosphorylation of JAK3 and STAT3 was retested. LMP1-siRNA2 showed stronger inhibition of LMP1 expression and was chosen for use in subsequent experiments (Figure 6C). Knockdown of LMP1 expression attenuated the inhibition of phosphorylation of JAK3 and STAT3 (SNK6-LMP1^Si^), whereas substantial reduction of phosphorylation of JAK3 and STAT3 was still observed in the control siRNA group (SNK6-LMP1^Scr^) (Figure 6D and 6E). The above results suggest that LMP1-IgG exerted tumor-inhibitory function by affecting JAK3/STAT3 activity.

## DISCUSSION

Antibody-based therapy is one of the most important strategies for cancer treatment, and a number of antibody-based drugs are undergoing clinical trials [19]. In our previous researches, we have identified several oncogenic biomarkers and produced related engineered antibodies through phage display technology [20, 21], including anti-LMP1 Fab [16, 17], anti-Trop2 Fab [22, 23], anti-MAGE-A1 scFv [24] and anti-hWAPL scFv [25]. However, a significant disadvantage is that phage display technology can-not construct full-length antibodies [26], but only antibody fragments (including Fab and scFv) that must be further transformed into whole IgG molecules to perform functions. Several disadvantages of antibody fragments remains to be further studied and resolved, such as the low absolute tissue uptake of Fab and the high degradation of scFv by proteases [27, 28]. In comparison, full-length IgG antibodies provide substantial benefits, such as increasing half-life in the circulation system, stimulating the complement system and engaging Fc receptor-mediated effector functions [29].

In this present study, we first constructed an LMP1-IgG antibody, on the basis of the previous LMP1-Fab. Eukaryotic expression vectors were prepared, and LMP1-IgG expression vectors were subsequently produced by IF-PCR. Then we successfully transfected the recombinant vectors and purified the LMP1-IgG, which was confirmed by a series of characterization experiments. Moreover, we tested the efficacy of LMP1- IgG at labeling LMP1 in ENKTL by IHC analysis. Compared with C-LMP1, LMP1-IgG displayed an equivalent ability to identify positive LMP1 ENKTL cells, thus further verifying the characteristics of LMP-IgG. Similar protocols were used in our previous study to investigate the function of LMP1-Fab in nasopharyngeal carcinoma (NPC) diagnosis [30].

Because LMP1-Fab has exhibited tumor-inhibitory potentiality in NPC [16, 17] and LMP1 plays important roles in ENKTL progression [9, 14, 15], whole LMP1-IgG is theoretically supposed to exert more significant anti-tumor effectiveness in ENKTL. Bearing this in mind, we performed a series of experiments to detect the tumor-inhibitory role of LMP1-IgG *in vitro*. The results of CCK-8 and MTT assays demonstrated that LMP1-IgG was able to inhibit the proliferation of SNK6 and SNT8 cells in a dose- and time-dependent manner. Moreover, the data of Annexin V/PI showed that with an increase of concentration and duration of LMP1-IgG in ENKTL cells, the apoptosis rate of SNK6 and SNT8 cells also elevated. LMP1 is widely acknowledged to act as an important oncoprotein that modulates several signaling pathways, including the nuclear factor kappa B (NF-κB), c-Jun N-terminal kinase (JNK), and phosphatidylinositol 3-kinase(PI3K) signaling pathways, thus promoting cell growth and suppressing apoptosis [31, 32]. In NPC for example, LMP1 preserves the cancer stemness of NPC cells by activating the PI3K/AKT pathway [33]; LMP1 critically mediates transformation of nasopharyngeal epithelial cells and facilitates FGF2/FGFR1 signaling activation in the EBV-driven pathogenesis of NPC [34]. In lymphoma, LMP1 protects lymphoma cells from cell death through the collagen-mediated activation of a receptor tyrosine kinase and makes an important contribution to promote the oncogenic effects of EBV [35]; LMP1 has also been reported to aggravate malignant cell function, induce surviving expression and inhibit cell apoptosis through NF-κB and PI3K/Akt signaling pathways [9, 36]. All the above data support that LMP1-IgG demonstrates dramatic ability to inhibit cell growth and accelerate cell apoptosis.

For therapeutic antibodies, ADCC and CDC have been proven to be important modes of action [37]. Recent studies have reported that IgG can activate component C1 through hexamers and lead to target cell killing by CDC via membrane attack complexes [38]. IgG amino acid residues that modulate FcγR binding can be modified to promote ADCC [39]. It is rational to hypothesize that ADCC and CDC may also play imperative roles in the tumor-inhibitory effectiveness of LMP1-IgG in ENKTL. The following data validated our presumption that LMP1-IgG would successfully activates ADCC and CDC in SNK6 and SNT8 cells, although the levels of ADCC and CDC varied in the two ENKTL cell lines. Several recent studies have described similar functions of antibody drugs in cancer therapy [40, 41].

The JAK/STAT pathway is crucial in signaling by cytokine receptors, blood formation and the immune response [42]. Consequently, it is well acknowledged that the JAK/STAT pathway plays tremendous roles in oncogenesis, including lymphomagenesis [43, 44]. Bouchekioua et al. have reported that activation of the JAK3/STAT3 pathway exerts major activity in ENKTL cell growth and survival; whereas tumor growth could be significantly suppressed by a JAK inhibitor [45]. Similarly, Coppo et al. have described that STAT3 activation is constitutively triggered and that the oncogenic STAT3 protein has important functions in the oncogenic process of ENKTL [46]. Because LMP1 has been stated to directly activate the JAK/STAT pathway, which is critical for ENKTL development [47, 48], we inevitably assumed that LMP1-IgG might display ENKTL-inhibitory characteristics by interfering with the JAK/STAT pathway. Therefore, we tested the effect of LMP1-IgG on the JAK/STAT pathway in ENKTL. The results showed that LMP1-IgG significantly inhibited phosphorylation of STAT3 but barely influenced the phosphorylation of STAT5. In comparison, the expression levels of STAT3 and STAT5 were stable after LMP1-IgG treatment. Subsequently, we continued to evaluate the effect of LMP1-IgG on signaling upstream of STAT. The data revealed that LMP1-IgG further blocked the phosphorylation of JAK3 but had no effect on the phosphorylation of JAK1, JAK2 or TYK2. Then, we explored whether LMP1-IgG influences the JAK/STAT pathway through recognizing LMP1. We employed siRNA to decrease LMP1 expression in SNK6 cells. The results manifested that the inhibition of JAK3 and STAT3 phosphorylation was significantly reduced in the LMP1^Si^ group (SNK6-LMP1^Si^). In comparison, the inhibition of JAK3 and STAT3 phosphorylation was witnessed in the LMP1^Scr^ group (SNK6-LMP1^Scr^). The above data implied that disruption of the JAK3/STAT3 pathway might be one of the potential mechanisms for LMP1-IgG's ENKTL-inhibitory ability. Several studies’ conclusions lend further support to our results and have suggested that targeting the JAK/STAT pathway may be a promising strategy for ENKTL therapy [49–51].

Interestingly, a previous study has shown that LMP1 can activate JAK3, as well as the downstream targets include STAT5 [52]. However, we did not detect an activity of LMP1-IgG in phosphorylation of STAT5 or expression of STAT5. The inconsistent data may be a result of the differences in tumor types, antigen epitopes or antibodies used. Future research is required to confirm our findings.

In addition, one major disadvantage of this present study is that we were unable to produce *in vivo* results. We encountered several problems in xenograft construction in BALB/c nude mice as a model for ENKTL, and new *in vivo* experiments on NSG mice are ongoing. We intend to draft a new paper specifically concerning the *in vivo* effectiveness of LMP1-IgG in ENKTL with the latest updated data.

To sum up, this study herein illustrates that a novel neutralized human LMP1-IgG exerts potent antitumor ability in ENKTL by interfering with the JAK/STAT pathway. Our findings may provide a novel and promising strategy for targeted therapy in ENKTL.

## MATERIALS AND METHODS

### Cell lines and reagents

A human LMP1-Fab was generated and preserved in our lab [16]. Three ENTKL cell lines SNK6, SNT8 and YT were also preserved in our lab and enrolled in the present study [53, 54]. SNK6 and SNT8 are LMP1-positive, whereas YT is LMP1-negative [10, 55]. Anti-β-actin was purchased from Boster Co., Ltd. (Boster, Wuhan, China). All other antibodies were purchased from Abcam (Abcam, Cambridge, UK). The 293Free style cells and 293F expression culture medium were purchased from Invitrogen (Invitrogen, Carlsbad, CA, USA). Two eukaryotic expression vectors (pTH-VH and pTH-VK) were purchased from Invitrogen Co., Ltd. (San Diego, CA, USA). An In-FusionR HD Cloning Kit was purchased from Clontech (Clontech Japan, Tokyo, Japan). Fresh human serum and peripheral blood mononuclear cells (PBMCs) were donated of Dr. Yuan Mao. All study protocols followed the guidelines and were approved by the Human Research Ethics Committees of Nanjing Medical University.

### ENKTL patients and tissue samples

Tissue samples from 26 ENKTL patients for immunohistochemistry (IHC) analysis were enrolled as previously described [56].

### Construction, expression and purification of LMP1-IgG

LMP1-IgG variable regions of the heavy (VH) and light chains (VK) were first amplified by PCR using an LMP1-Fab clone as the template [17]. The primers for VH and VK of LMP1-IgG were designed according to the protocol of Infusion-PCR (IF-PCR) and the primers were as follows: V_H_ forward: 5′-GGT GTC CAC TCG CTA CAG GTG CAG CTG GTG-3′ and V_H_ reverse: 5′-GCC CTT GGT GGA TGC TGA GGA GAC GGT GAC-3′; V_K_ forward: 5′-ACA GAT GCC AGA TGC GAC ATC CAG ATG ACC-3′ and V_K_ reverse: 5′-TGC AGC CAC CGT ACG TTT GAT CTC CAG CTT-3′ (Table 1). Two eukaryotic expression vectors (pTH-VH and pTH-VK) were then digested [57] and VH and VK were separately cloned into linearized pTH-VH and pTH-VK vectors by IF-PCR with In-FusionR HD Cloning Kit. Then, the recombinant vectors were transfected into FreeStyle™ 293-F Cells (293F) with a FreeStyle™ 293 Expression System (Invitrogen). After transient transfection for 120 h, the cell supernatant was harvested and purified with a Hitrap protein A column (AKTA Purifier 100, GE, USA) as previously described [57].

**Table 1:** Summary of primer sequences

Primer types	Primer sequences
V_H_ forward	5′-GGT GTC CAC TCG CTA CAG GTG CAG CTG GTG-3′
V_H_ reverse	5′-GCC CTT GGT GGA TGC TGA GGA GAC GGT GAC-3′
V_K_ forward	5′-ACA GAT GCC AGA TGC GAC ATC CAG ATG ACC-3′
V_K_ reverse	5′-TGC AGC CAC CGT ACG TTT GAT CTC CAG CTT-3′
LMP1-siRNA1 forward	5′-GGA AUU UGC ACG GAC AGG CTT-3′
LMP1-siRNA1 reverse	5′-GCC UGU CCG UGC AAA UUC CTT-3′
LMP1-siRNA2 forward	5′-GCU CUC UAU CUA CAA A-3′
LMP1-siRNA2 reverse	5′-UUU GUU GUA GAU AGA GAG C-3′
LMP1-control-siRNA forward	5′-UUC UCC GAA CGU GUC ACG UTT-3′
LMP1-control-siRNA reverse	5′-ACG UGA CAC GUUCGG AGA ATT-3′

### Characterization of LMP1-IgG

ELISA, western blotting (WB), and affinity assays (BiaCoreX100) for investigating the characteristics of LMP1-IgG were performed as previously described [22, 57].

### IHC analysis

IHC analysis was further performed to verify the function of LMP1-IgG to identify ENKTL samples. The protocol of IHC analysis was as described previously [58–65].

### Cell viability and apoptosis assays

Cell viability was tested by using CCK-8 (Dojindo Laboratories, Japan) and MTT assays following the manufacturer's instructions. Cell apoptosis was investigated by Annexin V/PI as previously described [22, 66]. An unrelated-IgG was employed as a control antibody.

### ADCC and CDC assays

For the ADCC assay, ENKTL cells (target cells) were incubated with LMP1-IgG. An unrelated IgG was used as the control. PBMCs were used (effector cells) and incubated with ENKTL cells at a fixed effector/target ratio of 25:1. After a 4-hour incubation at 37°C, the cell supernatants were added to a 96-well plate to evaluate LDH release by LDH Cytotoxicity Assay Kit (Beyotime, Shanghai, China). For the CDC assay, ENKTL cells were incubated with LMP1-IgG, and this was followed by the addition of 20% human serum or heat-inactivated human serum. Then, the cell supernatants were added to a 96-well plate to evaluate LDH release with a LDH Cytotoxicity Assay Kit (Beyotime). Both assays were performed according to the manufacturer's instructions [67].

### Small interfering RNA (siRNA) transfection

Two LMP1-siRNAs and a control-scrambled siRNA were chemically synthesized by Sigma Chemical Co., Ltd. (St. Louis, MO, USA). The sequences of the control-siRNA and LMP1-siRNAs were as follows: control siRNA forward 5′-UUC UCC GAA CGU GUC ACG UTT-3′, reverse 5′-ACG UGA CAC GUUCGG AGA ATT-3′; LMP1-siRNA1 forward 5′-GGA AUU UGC ACG GAC AGG CTT-3′, reverse 5′-GCC UGU CCG UGC AAA UUC CTT-3′; LMP1-siRNA2 forward 5′-GCU CUC UAU CUA CAA A-3′, reverse 5′-UUU GUU GUA GAU AGA GAG C-3′ (Table 1).

### The influence of LMP1-IgG on the JAK/STAT signaling pathway

WB analyses of the JAK/STAT signaling pathway were performed as previously described [22]. The JAK/STAT signaling pathway was investigated in SNK6 cells with LMP1-siRNA transfection (SNK6-LMP1^siRNA^) and scrambled LMP1-siRNA transfection (SNK6-LMP1^scr^) after treatment with LMP1-IgG.
